# Conditional Knockout of NMDA Receptors in Dopamine Neurons Prevents Nicotine-Conditioned Place Preference

**DOI:** 10.1371/journal.pone.0008616

**Published:** 2010-01-07

**Authors:** Lei Phillip Wang, Fei Li, Xiaoming Shen, Joe Z. Tsien

**Affiliations:** 1 Brain and Behavior Discovery Institute and Department of Neurology, School of Medicine, Medical College of Georgia, Augusta, Georgia, United States of America; 2 Yunnan Xishuang Banna Primate Model Research Center, Xishuang Banna, Yunnan, China; 3 Shanghai Children's Medical Center, Shanghai Jiaotong University Medical School, Shanghai, China; Duke University, United States of America

## Abstract

Nicotine from smoking tobacco produces one of the most common forms of addictive behavior and has major societal and health consequences. It is known that nicotine triggers tobacco addiction by activating nicotine acetylcholine receptors (nAChRs) in the midbrain dopaminergic reward system, primarily via the ventral tegmental area. Heterogeneity of cell populations in the region has made it difficult for pharmacology-based analyses to precisely assess the functional significance of glutamatergic inputs to dopamine neurons in nicotine addiction. By generating dopamine neuron-specific NR1 knockout mice using cre/loxP-mediated method, we demonstrate that genetic inactivation of the NMDA receptors in ventral tegmental area dopamine neurons selectively prevents nicotine-conditioned place preference. Interestingly, the mutant mice exhibit normal performances in the conditioned place aversion induced by aversive air puffs. Therefore, this selective effect on addictive drug-induced reinforcement behavior suggests that NMDA receptors in the dopamine neurons are critical for the development of nicotine addiction.

## Introduction

Nicotine from smoking tobacco produces one of the most common forms of addictive behavior. It is well established that systemic nicotine stimulates nicotinic acetylcholine receptors (nAChRs) in the ventral tegmental area (VTA), consequently enhancing the phasic firing of VTA dopamine neurons and the release of dopamine to targets including the nucleus accumbens. At a concentration close to those experienced by a cigarette smoker nicotine can modulate DA neuron firing by acting on both postsynaptic and presynaptic neuronal nAChRs. Activation of the postsynaptic nAChRs which locate on the DA neurons and are mainly β2 subunits containing[1], [2] depolarizes the DA neurons and in the mean time quickly leads to desensitization of the activated nAChRs. The presynaptic nAChRs which locate on the excitatory glutamatergic terminals that project onto the DA neurons and are mainly α7 subunits containing and highly permeable to calcium[3]. Activation of the presynaptic α7 containing nAChRs increases calcium in glutamatergic presynaptic terminals and facilitates glutamate release and subsequent synaptic excitation of DA neurons[4]. This presynaptic mechanism, when paired with post-synaptic depolarization, can induce long-term potentiation (LTP) [3] which greatly outlasts the activation of nAChRs. Similar to that seen in hippocampal Schaffer collateral pathway, induction of this LTP depends on functional NMDA receptors. Recruiting changes in a long-lasting glutamatergic signaling has been postulated to be crucial for explaining the time scale discrepancy between fast desensitization (within a few seconds) of nAChR after nicotine binding[5] and the often observed long-lasting (more than hours) reward/reinforcement effects of nicotine on addictive behavior[6]. Additionally, injection of NMDA receptor antagonists into the VTA blocks nicotine-induced burst firing of VTA dopamine neurons as well as prolonged dopamine release in the nucleus accumbens [6].

NMDARs are heteromeric assemblies of NR1, NR2 and NR3 subunits, which co-translationally assemble in the endoplasmic reticulum to form functional channels. NR1 subunits bind the co-agonist glycine and NR2 subunits bind the neurotransmitter glutamate. Activation of the NMDA receptor or opening of the ion channel allows flow of Na^+^ and Ca^2+^ ions into the cell, and K^+^ out of the cell[7]. Because the channel opening requires both presynaptic binding of glutamate and postsynaptic depolarization which removes Magnesium blockage, NMDA receptors function in many forms of synaptic plasticity as the coincidence detector. Furthermore, NR2 subunits of NMDA receptors which regulate channel properties such as conductance and opening duration have been suggested to contribute differently in LTP and LTD in the Shaffer collateral pathway[8], [9], [10], [11]. NR1 subunit of NMDA receptors is encoded by a single gene with several different splicing isoforms [12]. This subunit is essential for NMDA channel functions as deletion of NR1 completely abolished NMDA receptor responses[13], [14].

The NMDA receptors in the cortex and hippocampus are well known to be essential for normal forms of learning and memory [15]. *In vitro* brain slice studies show that bath application of nicotine enhanced NMDA receptor-dependent LTP[3]. This would pose the NMDA receptor as a strong candidate how nicotine modulates and hijacks the endogenous machinery where cellular association integrates glutamatergic signaling from the cortical and hippocampal regions with the dopamine reward pathways [16]. However, precise evaluations of the behavioral significance of glutamatergic inputs to DA neurons is hampered by the heterogeneity of VTA cell types. While 60∼70% of neurons in the VTA are dopaminergic, the diverse populations of other types of non-DA neurons[17], both excitatory and inhibitory, make it rather difficult for pharmacological approaches to precisely pinpoint the behavioral contributions of the dopamine neuron NMDA receptors on nicotine-induced addiction.

To probe the role of the NMDA receptors in drug addiction, we generated cre/loxP-mediated conditional knockout mice (DA-NR1-KO) in which the NR1 gene, encoding the core subunit for NMDA receptors, is deleted specifically in dopamine cells. With the DA neuron selective knockout of NR1 subunit which would completely abolish the NMDA receptor channel function in those cells, we investigated whether this genetic lesion impacts animals' abilities to form nicotine addiction. Additionally, we investigated whether the same mutation affects animals' context learning.

## Results

### Generation and histochemical characterization of DA-NR1-KO mice

**Figure 1:**
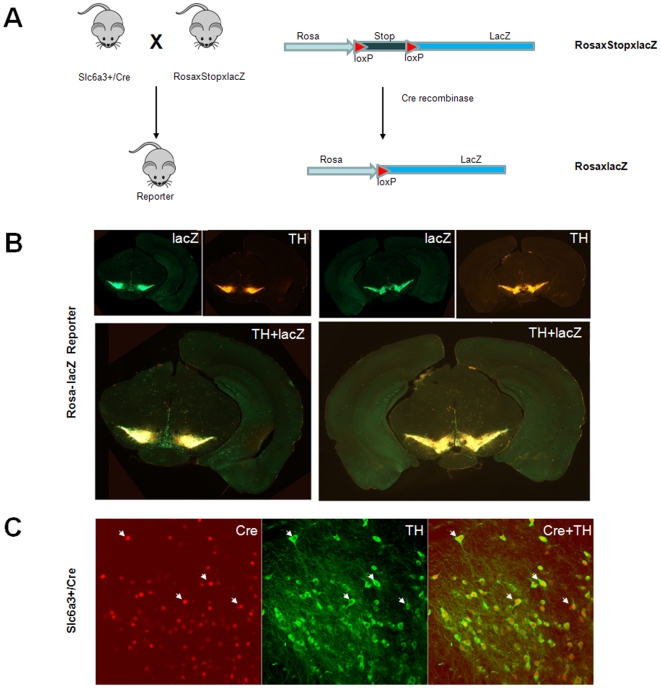
Generation of the reporter mouse and and histological characterization of Slc6a-cre induced deletion. (**a**) Breeding scheme for the production of the dopamine-neuron specific Rosa-stop-lacZ reporter mice. The dopamine neuron-specific Cre line (Slc6a3+/Cre) line was crossed with the rosaxstopxlacZ mic where “x” stands for the loxP sites. Deletion of the floxed stop sequence by cre recmbinase results in lacZ expression driven be the ubiquitously active rosa26 promoter. (**b**) Low-power (2×) images of immunofluoresence of two different coronal sections for lacZ and TH in the midbrain of the Rosa26-lacZ reporter mice, showing specific gene deletion in the VTA and substantia nigra. (**c**) High-power (10×) image of double immunofluoresence for TH (shown in green) and Cre recombinase (shown in red) in VTA dopamine neurons of the Slc6a3+/Cre mice. Cre positive neurons pointed by the arrows were also TH positive.

**Figure 2:**
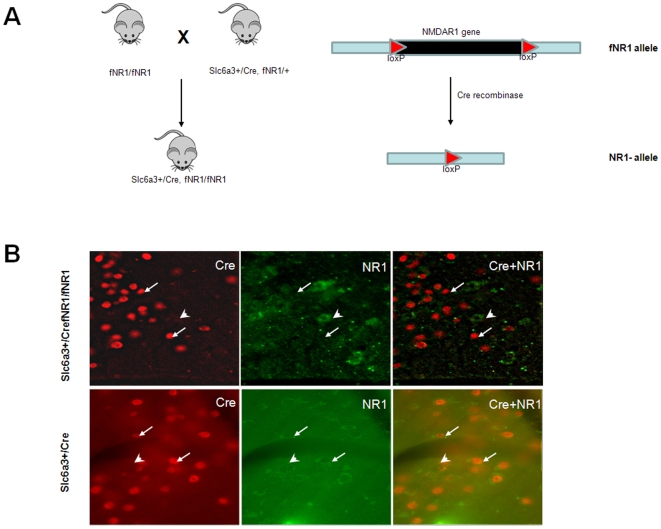
Generation and characterization of the DA-NR1-KO mice. (**a**) Breeding scheme for the production of the dopamine-neuron specific NR1 knockout mice. The dopamine neuron-specific Cre line (Slc6a3+/Cre) line was crossed with the floxed NR1 mice for two generations to produce the conditional knockout mice (Slc6a3+/Cre fNR1/fNR1), or DA-NR1-KO for short. Three groups of littermate mice were used as the controls: Slc6a3+/Cre fNR1/+, Slc6a3+/Cre alone, and wild type littermates. The genotyping was done as previously described^7,8^. (**b**) High-power (20×) image of immunofluoresence for Cre recombinase (shown in red) and fluorescent in situ hybridization for NR1 mRNA (shown in green) in VTA dopamine neurons. NR1 expression was abolished in Cre-positive (arrows), but present in TH-negative (arrow heads) neurons in the VTA of DA-NR1-KO mice.

**Figure 3:**
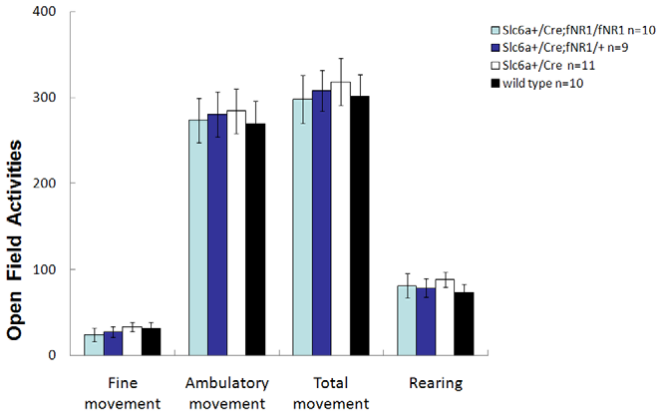
DA-NR1-KO mice display normal locomotor activities in a novel open field. Locomotor activity was measured as beam breaks in activity chambers (San Diego Instruments, San Diego, CA). Movements including the ambulatory movement, fine movement and rearing activities were scored based on beam break patterns. Mice were placed into standard sized rat cages which they have never been exposed to. Activities were monitored for 120 minutes. There was no difference between genotypes in tested animals' activities. (Post-Hoc test, P>0.05). Data were calculated as Mean ± SEM. ANOVA analysis (either repeated measure ANOVA or Post-Hoc test or Paired-t test, as appropriated) was used to determine the difference among groups.

The conditional knockout mice were produced by crossing the floxed NR1 mice [14] with the dopamine neuron-specific Cre mice, namely, Slc6a3-Cre mice (Figure 1A). In the Slc6a3-Cre mice, the Cre recombinase was driven by the endogenous Slc6a3 promoter [18]. We confirmed the dopamine neuron-specific gene deletion with a set of molecular genetic methods. First, cell type specificity of Slc6a3Cre-mediated gene deletion was revealed by lacZ expression when the Slc6a3-Cre mice were crossed with the Rosa-*loxP*-stop-*loxP*-lacZ reporter mice. It showed that gene deletion was restricted to the dopamine neuron regions such as the VTA and substantia nigra (Figure 1). Immunofluorescence showed highly identical patterns of lacZ and tyrosine hydroxylase (TH) expression (Figure 1B), verifying that the Cre/loxP-mediated gene deletion occurred exclusively in dopamine neurons. This is further supported by immunofluorescence for Cre recombinase and TH in the Slc6a3 Cre mouse, where TH and Cre were shown to be expressed in the same cells (Figure 1C). Finally, immunofluorescence of Cre and fluorecent *in situ* hybridization of NR1 mRNA confirmed that NR1 was deleted in DA neurons in the DA-NR1-KO mutant mice. (Figure 2). Despite of the NR1 deletion, the mutant mice were normal in size, mobility, food intake, and reproduction abilities. They also displayed normal basal locomotor activities when measured in a novel open field (Figure 3), and normal anxiety levels when measured using an elevated plus maze test (data not shown).

### DA-NR1-KO mice are impaired in nicotine induced CPP

**Figure 4:**
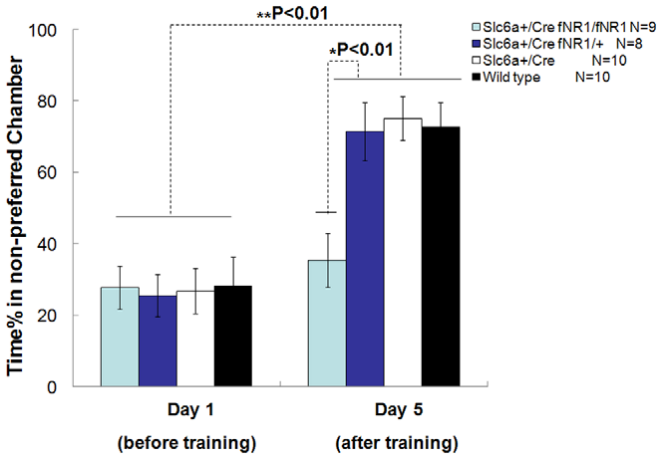
Selective prevention of nicotine-conditioned place preference in DA-NR1-KO mice. DA-NR1-KO mice failed to develop nicotine-induced conditioned place preference. The mice from all groups spent roughly 30% of total time in the non-preferred chamber before injection of nicotine or saline. After 4 consecutive days of counterbalanced injections of nicotine in the non-preferred chamber and saline in the preferred chamber, performance differences in genotype and interaction of genotype × day were revealed (Repeated Measure ANOVA, genotype F(3,33) = 5.52, P<0.01; interaction of genotype×day F(3,33) = 13.14, P<0.01). The three control groups of mouse each showed similarly strong preferences to the previously non-preferred chamber (Post-Hoc test, P<0.01 for the three groups, respectively). In contrast, the DA-NR1-KO mice did not show preference to the nicotine paired non-preferred chamber (Paired-t test, P>0.05). Post-hoc test showed significant difference between DA-NR1-KO mice and the control groups (P<0.01, respectively), but no difference among the three control groups. (P>0.05, respectively). Data were calculated as Mean ± SEM. ANOVA analysis (either repeated measure ANOVA or Post-Hoc test or Paired-t test, as appropriated) was used to determine the difference among groups.

We chose to use Conditioned Place Preference (CPP) to measure nicotine addiction. CPP measures the animals' tendency to stay in the chamber associated with rewards or safety (rather than the non-reward chamber). For testing the nicotine conditioned place preference in DA-NR1-KO mice, we employed an asymmetric contextual place preference apparatus and used three groups of littermates (Slc6a3cre alone, Slc6a3Cre with fNR1/+, and wild type) as controls. The conditioned place preference apparatus had two equal sized chambers, one with dark walls and a dark floor and other with white walls and a white floor. The two chambers also have different floor textures to further distinguish them contextually. A small holding cell was placed in the middle to also serve as the separator between the two chambers. Due to their natural tendency to avoid bright environments, mice showed significant initial preference to the dark chamber (preferred chamber) and spent significantly more time (about 70% of the total time) there when allowed to move freely prior to conditioning trials (Figure 4). There was no difference among genotypes, again indicating the normal amount of basal anxiety and fearfulness in the conditional knockout mice. To induce conditioned place preference, mice from the mutant and control groups were all injected with either nicotine (0.75 mg/kg of body weight) or with saline and then placed in one of the two chambers. Nicotine injections were paired with the non-preferred chamber and saline injections paired with the preferred chamber. After four days of conditioning, during which mice were given one injection of nicotine and one injection of saline each day (one in the morning and one in the afternoon in a counterbalanced manner), CPP was tested on the fifth day. Each mouse was allowed to move freely between all the chambers once the holding cell was lifted. The mice spent very little time in the holding area (which was very small in size). Time spent in the previously non-preferred and preferred chambers were recorded and the percentages of time spent in the non-preferred chamber were calculated and compared within and between the groups. After nicotine conditioning, all three control groups exhibited a strong preference toward the previously non-preferred chamber by spending a significantly increased amount of time in there (Figure 4, p<0.01). However, the DA-NR1-KO mice failed to develop the conditioned preference towards the nicotine injection-associated chamber when comparing with the control groups (p<0.01). This suggests that deletion of the NMDA receptors from dopamine neurons prevents the nicotine conditioned place preference.

### DA-NR1-KO mice display normal aversive stimulus induced CPA

**Figure 5:**
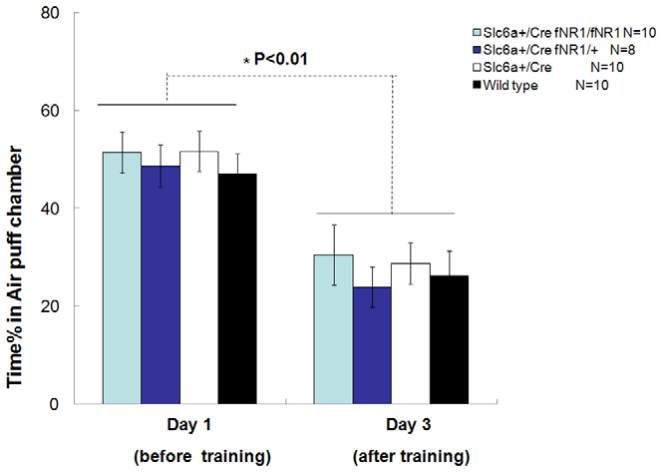
Normal air puff-induced conditioned place preference in the DA-NR1-KO mice. CPP was done using the symmetric conditioning chamber. The mice from all groups spent roughly 50% of total time in the air puff chamber before the air puff was delivered. After air puff (on day 1), when the mice were tested on day 3, the 48-hour retention tests revealed that all mice groups exhibited strong preference to the safe chamber and spent less time in the air puffed chamber (Paired-t test, P<0.01 for the three groups, respectively). Moreover, there was no difference between genotypes in their percentage of time spent in air puffed chamber among all the four groups tested (Post-Hoc test, P>0.05). Nor any difference in interaction of genotype×day was found (Repeated Measure ANOVA, genotype F(3,34) = 1.14, P>0.05; interaction of genotype×day F(3,34) = 0.14, P>0.05 ). Data were calculated as Mean ± SEM. ANOVA analysis (either repeated measure ANOVA or Post-Hoc test or Paired-t test, as appropriated) was used to determine the difference among groups.

Lack of nicotine conditioned place preference in the mutant mice could have resulted from either the genuine lack of nicotine addiction or mice's inability to recognize and remember the contextual cues. To distinguish these two possibilities, we measured conditioned place aversion induced by mild aversive signals in the mutant and three groups of control mice. In order to eliminate influence of nicotine exposure on CPA, new subjects were chosen which have not been previously tested for nicotine induced CPP. The CPA test was done using a symmetric testing apparatus consisting of two equal sized chambers of which mice showed similar preferences before conditioning as previously reported [19]. Specifically, we used multiple sudden air puffs as aversive stimuli to startle the mice. This protocol has been shown to be very effective in inducing robust place aversion memory in mice. Under this aversive stimulus-induced conditioning regiment, all three groups of the control mice exhibited a significant amount of place recognition memory (Figure 5, p<0.01). Interestingly, the mutant mice also developed comparable conditioned aversion of the air puffed chamber and learned to favor the non-air-puffed chamber (Figure 5, p<0.01). This indicates that the mutant mice preserved not only the normal preference/aversion memory (by recognizing the chamber associated with safety), but also the normal amounts of negative motivational salience to stay away from the unsafe chamber.

## Discussion

Using conditioned place preference as the behavioral readout, we studied nicotine induced associate learning in a genetically modified mouse model where NMDA receptor function is selectively blocked in the dompaminergic neurons. Comparing with their littermates, the mutant DA-NR1-KO mice, displayed clear deficits in developing nicotine conditioned place preference. In contrast, these mutant mice displayed normal learning and memory in air puff induced place aversion, a paradigm that shares some similar contextual memory components but differs in the nature of enforcement. These results therefore suggest that lack of nicotine mediated conditioning is likely due to reward-reinforcement components than context associative memory process. Because of the nature of the genetic lesion, a DA neuron selective deletion of NMDAR1, our data strongly suggest NMDAR1 mediated signals in DA neuron be an essential element for developing nicotine induced associative learning.

At the circuitry level, DA neurons receive robust glutamatergic inputs from brain structures such as the prefrontal cortex, subthalamic nucleus and hippocampus[16]. NMDA receptors are known to be a key element in integrating glutamatergic inputs. The importance of NMDA receptors in DA neurons is evident with all the addictive drugs induced LTPs found to dependent on NMDAR. Midbrain dopaminergic neurons, especially in the mesolimbic system has been implicated as major candidates to mediate incentive salience in the natural reward pathway. It has been postulated that via their extensive dopaminergic efferents to brain structures such as the hippocampus, striatum and prefrontal cortex, DA neurons regulate many forms of learning by modulating synaptic plasticity[20]. Addictive substance which are thought to work by hijacking the natural rewarding pathways often target DA neurons, with morphine, nicotine, ethanol, and either cocaine or amphetamine, drugs with little in common beyond their abuse potential, all able to induce LTP in DA neurons in VTA[21]. Furthermore, nearly all addictive drugs produce an acute increase in the release of dopamine from VTA neurons at their terminals in the nucleus accumbens.

Interestingly, nicotine is a unique and potent addictive substance because it binds postsynaptically to mainly β2 containing nAChRs to depolarize DA neurons, and presynaptically to α7 containing nAChRs to elevate glutamate release. The combined effects on both pre- and post-synaptic targets make nicotine an effective inducer of LTP in the DA neurons. The enhanced synaptic plasticity is NMDA receptor dependent thus presumably blocked in the mutant mice missing NMDAR1 in the dopaminergic neurons. The selective lose of abilities to obtain nicotine induced conditioning in these mice therefore not only vindicates NMDAR1 in DA neurons being essential to nicotine addiction but also strongly suggests that nicotine mediate its reward effects by modifying/enhancing synaptic plasticity of the excitatory glutamatergic efferents to DA neurons. It is quite conceivable that enhancing plasticity at glutamate inputs to DA neurons might represent a general mechanism for developing reward induced conditioning.

It is important to point out that besides DA neurons, GABAergic neurons in VTA which provide important regulation to DA neuron also express NMDAR1. The DA specific NMDAR1 deletion allowed us the isolate functions of NMDA receptors in DA neurons. But it would be of great interest to evaluate roles of these inhibitory neurons in addiction. Furthermore, nicotine was known to produce modulatory affects via nAChRs at axonal, perisynaptic and non-synaptic locations in many other brain areas such as hippocampus. In fact, studies have implicated the important roles of both hippocampus and nucleus accumbens in developing nicotine induced place preference[22], [23].

Finally, several recent studies have reported increased firings of some dopamine neurons by aversive stimuli such as air puff or tail shock [24], [25]. This raises an interesting question regarding how the positive and negative motivational salience values are processed in the VTA, and how the NMDA receptors play differential roles in these distinct representations. Nonetheless, the selective impairment of nicotine conditioned preference, but not the air puff-conditioned aversion suggests that there may be enough separation of normal memory vs. drug abuse memory within the cell population and the brain's neural circuits for processing motivationally distinct cues. In conclusion, our present study has provided some clear genetic evidence for the role of the dopamine neuron NMDA receptors in the initial phase of nicotine addiction.

## Materials and Methods

### Ethic statements

All animal work described in the study have been conducted according to NIH guidelines and approved by Institutional IACUC committee.

### Generation of DA-NR1-KO mice

Mice carrying an allele of NMDAR1 flanked by loxP sites (fNR1; “floxed” NR1) and were bred with Slc63a Cre transgenic mice. Offspring were genotyped by PCR for both the Cre transgene and for the floxed NMDAR1 (fNR1) allele. Mice used in these experiments have been bred for at least five generations onto the C57/BL6 background. Animals were maintained on a 12 hr light/dark cycle in the Medical College of Georgia animal care facility. Food and water were given ad libitum. All procedures relating to animal care and treatment conform to the Institutional and NIH guidelines. For behavioral tests in the study we used male mice around 1 year old in age. These animals have been prescreened to make sure that they have normal vision and hearing capacity.

### Immunocytochemistry

Mice were perfused transcardially with 4% paraformaldehyde (PFA) in 1x PBS followed by a post fixation in 4% paraformaldehyde overnight. Coronal sections (50 µm thick) were cut on a Vibratome and collected in 0.5% PFA in 1xPBS and stored at 4°C before use. For immunofluorescent staining, sections were incubated at 4°C overnight with gentle shaking in primary antibody [anti-Cre polyclonal antibody (pAb), 1/4000, BABCO; Anti-beta-Galactosidase (pAb) 1/5000, Invitrogene; anti tyrosine hydroxylase (TH) (monoclonal antibody) 1/1000) ]in a buffer containing 0.05% Tween20, 10% normal goat serum and 1x PBS following pre-incubation in 10% normal goat serum and 1× PBS at room temperature for 2 hours. The sections were then incubated with Alexa conjugated secondary antibodies (1/200, Invitrogene) at room temperature for 2 hours. Cre-IR was visualized by Alexa 568, TH-IR by Alexa 488 and lacZ-IR by Alexa 594. Fluorescent images were captured with a confocal laser scanning microscope and an epifluorecent microscope.

### *In situ* hybridization of NR1 mRNA

Coronal sections (50 µm in thickness) were cut on a Vibratome and collected in 0.5% PFA in 1xPBS. Hybridization was performed at 55°C for 18 hours in a hybridization buffer containing 50% formamide. Complementary RNA (cRNA), derived from the AvrII-SphI 0.4-kb antisense DNA fragment of rat NR1 cDNA containing from exon 13 to exon 16, was labeled with DIG (Roche) and used as the probe for detecting NMDAR1 mRNA in mouse brain. The brain sections were serially washed at 55°C with a set of SSC buffers of decreasing strength, with the final strength being 0.1 SSC. The sections were then incubated with HRP conjugated anti-DIG antibody in 10% normal goat serum in PBS at room temperature for 2 hours. Following washing, NR1 signal was detected with the TSA™ Kit (Invitrogene) using Alexa Fluor® 488 tyramide.

### Nicotine conditioned place preference

To test nicotine conditioned place preference, we employed an asymmetric contextual place preference apparatus which contains two equal sized conditioned chambers one with dark walls one with bright walls. The two chambers were separated by a small sized holding chambers which also served as the partition between the two conditioning chambers. The conditioned place preference paradigm took place on 5 consecutive days using the biased apparatus. Since animals preferred the dark chamber, nicotine injections were always associated with the non-preferred chamber and saline with the preferred chamber. Injections were counterbalanced and were 6 hours apart in any given day. Before conditioning on day 1, each mouse was placed separately in the apparatus for 10 min, with free access to both chambers. The conditioning procedure consisted of a 3-day schedule of double conditioning sessions (i.e. days 2–4). The first day involved a morning session (9:00–11:00 am) in which mice received a single intraperitoneal (i.p.) dose of nicotine and were placed immediately in the non-preferred chamber for 30 min. This compartment had been separated from the other using a removable holding cell. In the afternoon session (3:00–5:00 pm) the animals received a single injection of saline, and were placed for 30 min in the preferred chamber. On the second day of conditioning, the animals received the saline injections in the morning session and the drug administration in the evening session. The third day of conditioning had the same schedule as the first day. On the fifth day of the schedule, as before the conditioning, the holding cell was raised and the mice were placed in the apparatus (between white and black chambers) and allowed again to freely explore the two chambers for 10 min. The time spent in the white or black chambers was recorded for each mouse. The proportions of time spent by each mouse in the non-preferred chamber were calculated and used as the index for conditioned place preference.

### Air-puff conditioned place aversion

We employed a symmetric apparatus for testing air-puff conditioned place aversion as reported in our previous study. The mice were handled and habituated before experiments. On day 1, the mice were allowed to freely explore the environment of a two-chambered conditioned place preference apparatus (20 × 40 × 20 cm) for 10 min (pre-training). The amounts of time spent in each chambers were recorded. On day 2 (the training day), each mouse was conditioned in a 10 minute session during which sudden air blows were delivered whenever the mouse visited one of the randomly assigned chamber (termed air-puff chamber). The other unconditioned chamber was designated as the safe compartment. On day 3 (testing day), 24 hours after conditioning, place preference was measured by returning the mice to the place conditioning environment for 5 min. The time spent (sec) in the air puff chamber and the safe chamber was measured and the proportions of time spent by each mouse in the air-puff chamber were calculated and used as the place preference index.

### Open field activity test

Locomotor activity was measured by scoring beam breaks in activity chambers (San Diego Instruments, San Diego, CA). Prior to open field tests, animals were handled for two consecutive days. Standard rat cages were used as the novel open field for the mice tested.
